# The role of general dynamic coordination in the handwriting skills of children

**DOI:** 10.3389/fpsyg.2015.00580

**Published:** 2015-05-07

**Authors:** Andrea Scordella, Sergio Di Sano, Tiziana Aureli, Paola Cerratti, Vittore Verratti, Giorgio Fanò-Illic, Tiziana Pietrangelo

**Affiliations:** ^1^Dipartimento di Neuroscienze, Imaging e Scienze Cliniche, Università degli studi “G. d’Annunzio” Chieti–PescaraChieti, Italy; ^2^Laboratorio di Valutazione Funzionale, Università degli studi “G. d’Annunzio” Chieti–PescaraChieti, Italy; ^3^Interuniversity Institute of MyologyChieti, Italy

**Keywords:** motor quotient, handwriting, postural control, KTK test, visual–spatial skills

## Abstract

Difficulties in handwriting are often reported in children with developmental coordination disorder, and they represent an important element in the diagnosis. The present study was aimed at investigating the relation between motor coordination and handwriting skills, and to identify differences in handwriting between children without and with coordination difficulties. In particular, we asked whether visual–spatial skills have a role as mediating variables between motor coordination and handwriting. We assessed motor coordination as well as graphic abilities in children aged 7–10 years. Moreover, we evaluated their visual–motor integration, visual–spatial skills, and other cognitive abilities (memory and planning). We found no relation between motor coordination and handwriting skills, while visual–spatial skills (measured by a visual-constructive task) were related with both. Our conclusion is that visual–spatial skills are involved both in general motor coordination and in handwriting, but the relationship involves different aspects in the two cases.

## Introduction

Handwriting is a complex task that involves both visual–motor and cognitive skills (Rosenblum et al., 2010; Bara and Gentaz, 2011). This task is managed in the western literate community during primary school. Although children begin to integrate visual and proprioceptive information by carrying out the tasks of copying shapes and letters (Daly et al., 2003) when they are preschoolers, it is during the school age period that they learn to associate movements with the mental image of the letters, and to write from dictation, so becoming able to control the movements proactively (Meulenbroek and Van Galen, 1988). At the same time, practicing that ability during the school period allows the process of handwriting to become more and more automatic (Feder and Majnemer, 2007). The acquisition of handwriting also affects more advanced literacy skills, such as the ability to produce written texts, because children who are in trouble with the graphic design of the graphemes are also likely to have fewer resources for the planning of the text (Berninger et al., 1997).

Difficulties in handwriting are seen in children with developmental coordination disorder (DCD), although not all children with dysgraphia also have DCD (Chang and Yu, 2010). DCD (or specific developmental disorder of motor function) is a non-verbal learning disorder that typically occurs in the absence of an injury, neurological disease, or major sensory dysfunction, although it is often associated with other disorders, such as ‘learning disorders’ (Lazarus, 1990) and ‘attention deficit disorder’ (Kadesjö and Gillberg, 1998). DCD is characterized by poor abilities in daily activities that require motor coordination, and independent of chronological age and intellectual functioning, this thus causes the affected children to show poor motor coordination, clumsiness, lack of coordination, and visual–motor and psychomotor instability (Van der Meulen et al., 1991), along with deficits in fine motor coordination and perceptual integration (Goyen et al., 1998). Children with DCD also have specific difficulty in the somatic-centered perception of their own movements, which prevents them from moving easily in space, and can alter, in turn, the organization of their executive patterns for actions (Frascarelli and Corcelli, 2000).

At the clinical level, children with DCD show marked difficulties in the modulation of their movements, changes in posture, and execution of complex acts. Moreover, as shown by Cairney et al. (2011), they are at greater risk of being overweight and obese than their peers. They can also have language and writing difficulties (Rosenblum and Livneh-Zirinski, 2008; Prunty et al., 2013), and more in general, they can show associated attention problems, dysgraphia, and learning difficulties (Tseng et al., 2007). Difficulties in handwriting are typically associated with problems in fine motor skills, probably due to an increased level of neuromotor noise that is compensated for by the increased phasic stiffness of the limb system (Smits-Engelsman et al., 2001). Indeed, handwriting requires a high level of coordination and high precision–force regulation, and therefore it is reasonable to assume that this ability is affected by motor coordination impairment (Smits-Engelsman et al., 2001). As empirical support, several studies that have compared handwriting skills between children with DCD and typical children have shown that those with DCD write faster and in larger letters (Jolly and Gentaz, 2014). Moreover, children with coordination disorders have less redundancy in the strategies of movement and are less flexible in adapting to the constraints of the task (Smits-Engelsman et al., 2001).

What handwriting deficits do children with motor coordination problems have? According to the literature, children with DCD produce less written text, due not so much to the slow movement execution, but rather to a higher percentage of time spent pausing (Prunty et al., 2013; see also Rosenblum and Livneh-Zirinski, 2008 for the ‘pausing phenomenon’ in children with DCD). As a further support of the pausing influence, Prunty et al. (2014) recently showed that children with DCD had more pauses of more than 10 s and paused more within words than typical children, which indicates a lack of automaticity in their handwriting. Children with DCD also take longer to produce letter strokes across a range of handwriting tasks, including during copying (Rosenblum and Livneh-Zirinski, 2008; Chang and Yu, 2010), writing from memory (Chang and Yu, 2010), the habitual task of writing ones name (Rosenblum and Livneh-Zirinski, 2008), and 10 min of free-writing (Prunty et al., 2013). This pattern of difficulties appears to be a quite robust phenomenon, as it has been found in different languages, such as Chinese, Hebrew, English, and French (Rosenblum and Livneh-Zirinski, 2008; Chang and Yu, 2010; Prunty et al., 2013; Jolly and Gentaz, 2014).

As DCD involves the abilities of planning, coordinating, and controlling movements, it is present also in dyspraxic children, who appear to have two main problems: difficulties in integrating sensory information from different senses (e.g., sight, touch, kinesthetics, hearing), and as a consequence, difficulties in organizing a well-defined plan of action (Sabbadini, 2005). Although several studies have shown a relationship between general coordination skills and reading-related measures (Francks et al., 2003), the relationship between general motor coordination and handwriting remains poorly investigated. In this case, most studies have been focused purely on aspects concerning the fine motor skills (Tseng and Chow, 2000; Feder and Majnemer, 2007).

Moreover, a handwriting deficit could be originating, at least in part, from a lower level deficit involving both a visual attention deficit (Franceschini et al., 2012, 2013) and/or even a magnocellular-dorsal deficit (Gori et al., 2014a) as it was found in dyslexia (Gori et al., 2014b; Gori and Facoetti, 2015). A lower deficit in the primary process of rapidly processing visual stimuli, could produce a cascade effect on the abilities of these children in handwriting.

The present study was designed to investigate the possible influence of motor coordination on graphic gesture patterns and the underlying processes. We assessed the motor coordination in children of 7–10 years of age, as well as their graphic abilities in a writing task. Moreover, we evaluated their general dynamic coordination and the visual–perceptual components, as related to the fine motor skills involved in the graphic gesture patterns. We were also interested to investigate the relations between coordination, postural control, and body mass index (BMI). We also controlled for the anthropometric parameter as the BMI, due to its influence in motor coordination.

The first hypothesis involves the relationship between motor coordination, as measured by the motor quotient (MQ), and balance control, as measured by stabilometric parameters, such as the Romberg Index (RI). Balance control involves proprioceptive, vestibular, and visual systems, and it is reached in different ways during development. Indeed, as they grow older, children tend to make less use of visual feedback, and to rely more on proprioceptive feedback and anticipatory control of their movements (Feder and Majnemer, 2007). The RI indicates an increase in fluctuations under the eyes-closed condition. In other words, the greater the difficulty they have in controlling their balance under the eyes-closed condition, the higher their RI. Therefore, we expect that children with a lower MQ would also have a higher RI.

The second hypothesis involves the relationships between MQ and visual–motor integration (VMI; as measured by the VMI test), expecting that children with a higher MQ will also have higher scores on visual–motor integration tasks.

The third hypothesis involves the relationships between MQ and visual–spatial skills, memory, and planning. The visual–spatial skills (particularly visual constructive) are involved in motor coordination in general, as motor coordination involves the ability to move in space using different coordinate systems and exploiting the processes of mental rotation. As motor skills allow relevant elements from the external environment to be perceived in order to regulate a movement in progress, they might affect planning of movement sequences in complex tasks to decide as quickly as possible the most effective action for a given target. Therefore, we expect that children with higher MQ also have higher values for the above abilities, compared to children with low MQ.

The fourth hypothesis concerns the relationship between general motor coordination and handwriting, with respect to selected aspects, such as speed, metric spatial variation (character height and fluctuation on the line), and legibility of letters. We expected that children with a lower MQ will have poorer handwriting performance. Moreover, we expected that visual–spatial skills are involved both in motor control (third hypothesis) and in handwriting, and have a role in this process as a mediating factor between them.

## Participants, Materials, and Methods

### Participants

The participants were recruited in three different schools, after permission was granted from the person responsible for the schools. The 84 children assessed were between 7 and 10 years of age (mean age, 8.5 ± 1.1 years), and comprised 42 males (mean age, 8.5 ± 1.0 years) and 42 females (mean age, 8.5 ± 1.1 years). One of these male children was excluded from the study due to his motor disability. Of the males, we analyzed 24 in the second year of primary school (class II; mean age, 7.6 ± 0.36 years), and 17 in the fourth year (class IV; 9.5 ± 0.30 years). Similarly, for the females there were 25 in class II (7.5 ± 0.38 years) and 17 in class IV (9.4 ± 0.30 years).

At the procedural level, we first performed interviews with the parents of the selected children, about the motor history of the children, the type of sport practiced, the weekly frequency and duration of each sports session, as well as about their possible diagnosed developmental disorders. Only one child presented motor disability. None of the children recruited was affected by disorders that could interfere with the tasks submitted. The children were then tested at school, by the psychologists and physiologists, in the presence of the teachers, who participated to the project. The tests were submitted in different days/sessions. The BMI was calculated according to their measured weight (kg) and height (m), specifically as the weight divided by the height squared.

The study was approved by the Ethics Committee of the ‘G. D’Annunzio’ University of Chieti–Pescara, and informed consent was signed by the parents of the children. The study conformed to the Declaration of Helsinki.

### Body Coordination Test

The *Korperkoordinations Test fur Kinder* (*KTK* test; *Coordination Test for Children*) by Kiphard and Schilling (1974, 2007) is a test that has been validated for analysis of motor coordination of children from 5 to 14 years of age. This test measures the dynamic coordination and motor control of the body, and it is appropriate for children with a pattern of normal development, as well as for children with brain damage, behavioral problems, and learning difficulties. The *KTK* test aims to highlight the presence of motor deficits in children, and focuses on the analysis of balance, rhythm, laterality, speed, and agility. The test consists of four tasks: balance (MQ1), to evaluate the stability of balance in the forward and backward paths; hopping on one leg over an obstacle (MQ2), for coordination of the lower limbs and their dynamic power/force; jumping laterally (MQ3), for the speed of execution with alternating jumps; and shifting platforms (MQ4), to test their laterality and space–time structure. At the end of the tasks, the operator sums the four results to obtain the MQ, which is normalized according to gender and age.

The MQ is considered an index of motor performance, which can be grouped as: MQ <56, impossible (MQI); MQ 56–70, severe motor disorders (<5th percentile; MQS); MQ 71–85, mild motor dysfunction (<15th percentile; MQMD); MQ 86–115, normal (16–84th percentile; MQN); MQ 116–130, good (>85th percentile; MQG); MQ 131–145, high (MQH). We considered the differences among the MQMD and MQN+MQG groups, due to our specific interest for the lower side of distribution compared to the rest of children.

### Stabilometric Test

We measured the child’s ability to integrate the visual–motor skills and balance postural sway by using the stabilometric–baropodometric platform equipped with the Physical Gait software. This test allows the recording of information related to the center of pressure (COP). Posture was measured under two different sensory conditions: looking straight ahead, with open eyes (OE) and with closed eyes (CE). This vision suppression can be used to estimate the importance of such a source of information in postural control, and to infer how the central nervous system adapts and reorganizes under postural sway.

The experimental sessions were composed of two balance tests, one for each sensory condition (i.e., OE, CE), with each trial lasting 30 s, to provide reliable measures relating to the postural sway (Le Clair and Riach, 1996). The reliability of the intra-class correlation coefficients of our parameters in our experimental session was 0.8 (*p* < 0.0001), in agreement with the finding reported by Geldhof et al. (2006) for similar methods.

The sequence of tests was interspersed with 1-min rest periods between the conditions, to avoid effects of learning or fatigue. Participants were asked to stand barefoot and in silence, with their feet at an angle of 30°, on a force platform that was incorporated into the ground (Physical Gait software, baropodometric and posturographic, with 4,800 active electronic sensors on a surface area of 120 cm × 320 cm).

During the trial with the OE conditions, the children were standing and staring straight ahead at a 3-m-away target, although they were not required to fix their vision on any particular point. To obtain a quantitative description of the ability to balance, we measured the following parameters of the COP: (i) the surface of the displacement of the COP on the *XY* plane (COP–SD), as the measure of the dispersion of the oscillations on the supporting plane, expressed in mm^2^ (Chiari et al., 2000); (ii) the RI, as the relationship between the individual ellipse surfaces obtained from the CE and OE analyses, expressed in perceptual values. This represents the surface of the ellipse of confidence that contains 90% of the sampled positions of the COP (Takagi et al., 1985; De Araújo Paloma et al., 2014).

The stabilometric test expresses the effectiveness of the postural system to maintain the center of gravity close to its average position of equilibrium. These data are reported as the median, first and third quartiles, and minimum and maximum, and mean with SE.

### Assessment of Visual Motor Integration

The *VMI* test (*Beery–Buktenica Developmental Test of Visual–Motor Integration*, Beery and Buktenica, 2000) is a paper and pencil test in which the child has to copy geometric forms that become progressively more difficult. The aim is to measure the VMI, as the ability to control hand movements guided by vision. The data were analyzed using standard scores.

### Assessment of Cognitive Abilities

The *Kaufman Assessment Battery for Children*, second edition (*KABC-II*; Kaufman and Kaufman, 2010) is a battery of tests that measures the cognitive abilities and processes based on two theoretical models of how our minds work: the psychometric model of specific cognitive abilities of Cattel–Horn–Carroll, and the information processing of the neuropsychological model of Luria. The complete battery includes 18 tasks, and here we administered six tasks related to the abilities we hypothesized to relate to motor coordination (see the fourth hypothesis in the Introduction) grouped according to three subtests related to sequential (memory), planning (reasoning), and simultaneous (visual–spatial) processes, according to the age of the children, as a scaled score:

(1) Sequential processes: (i) Number Recall: the child has to repeat a series of numbers in the same order in which the psychologist says them; (ii) Word Order: respecting the order given by the examiner, the child has to indicate the figures in a series of drawings that represent objects.
(2) Planning processes: (i) Pattern Reasoning: the child has to complete the set of a series of stimuli that form a logical sequence by choosing the correct item among those proposed; (ii) Story Completion: the child has to choose the picture that completes a story that is presented in pictures, where some of the pictures are missing.
(3) Simultaneous processes: (i) Rover: the child has to move a toy dog on a piece of cardboard divided into little squares while looking for the fastest route to reach the destination using the lowest number of movements; (ii) Triangles: the child has to assemble some triangles to form a figure.

The data were analyzed using standard scores, both for the three processes and for the six tasks. We were especially interested in the Triangles task as a measure of visual–spatial skill.

### Assessment of Handwriting

Two tests were used to assess both quality and efficiency of handwriting: the *Praxis subtest of BVSCO-2* and the DGM-P.

#### (a) Praxis of Writing

Praxis of handwriting was examined using *BVSCO-2* (*Batteria per la Valutazione della Scrittura e della Competenza Ortografica*-2; *Test for the Evaluation of Writing and Orthographic Ability*), according to three handwriting tasks: (a) writing the sequence of letters “LE” (handwritten lowercase cursive characters) for 1 min (LE praxis); (b) writing the sequence of letters “UNO” (ONE) for 1 min (UNO praxis); (c) writing the sequence of numbers UNO-DUE-, and so on (ONE–TWO–,…) for 1 min (Number praxis). The test involves the calculation of the measure of fluency: how many graphemes are written correctly in 1 min. The data were analyzed to provide the *z* score.

#### (b) Grapho-Motor Difficulties in Writing

The *DGM-P* (*Difficoltà Grafo-Motorie e Posturali della scrittura*; *Grapho-Motor, and Postural Difficulties in writing*) is an assessment tool for grapho-motor and postural difficulties in writing (Borean et al., 2012). The DGM allows an assessment to be obtained of the cursive handwriting in children from second to fifth grade of primary school. The test requires to perform in succession two transcripts in cursive of a simple sentence written in lowercase letters, according to two different conditions, one that focuses on accuracy (Best writing), and the other that focuses on the speed (Fast writing) of handwriting. The analysis is based on the quantification of 12 variables (indices or parameters) that characterize the efficiency or inefficiency of the handwriting, allowing information to be obtained with respect the speed of execution of the task and the readability of the written text.

We measured 10 performance indices under the Best writing condition: (i) Speed; (ii) Self-Correction; (iii) Fluctuating Letters; (iv) Dysmetria; (v) confusion between similar letters (Confusion Letter); (vi) Size of ascending and descending letter segments; (vii) unrecognizable letters; (viii) maximum amplitude of fluctuation between letters (Amplitude Fluctuation); (ix) maximum variation in the height of mean letters; (x) maximum variation in the height of ascending and descending letters.

We grouped the 10 measures into three categories: (a) speed (Speed index); (b) Spatial metric variation (maximum amplitude of the fluctuation between letters, maximum variation in the height of mean letters, maximum variation in the height of ascending and descending letter indices); (c) legibility of letters (Self-correction, Fluctuating Letters, Dysmetria, Confusion between similar letters, Size of ascending and descending letter segments, unrecognizable letters indices). The speed is given as letters written per second, the spatial metric variation is given in mm, and the legibility of letters involves counting the number of letters for each parameter. The speed and spatial metric variation were normally distributed, so we could make the transformation into *z* scores and combine the measures for metric variation. The measures of legibility of letters were not normally distributed, so we decided to find a cut-off for bad handwriters: people with at least two (of six) measures with a score equal to or less than the fifth percentile were classified as “bad handwriters.”

### Statistical Analysis

Statistical analysis was performed using the GraphPad Prism software, version 5 (GraphPad Software, La Jolla, CA, USA) and IBM SPSS Statistics version 20. Statistical comparisons were calculated using Pearson correlations, 95% confidence limits, and as two tailed. Comparisons were also made at the individual level by distinguishing MQMD as the clinical group, and the combined MQN+MQG children as the typical group. The Grubbs’ test, α < 0.05, was used to detect the outliers (http://www.graphpad.com/quickcalcs). We eliminated seven outlier values in the stabilometric measurements derived from three different subjects belonging to MQN+G category (COP–SD, OE and CE, and RI values of two subjects and RI value for one third subject). The one tailed Mann–Whitney non-parametric tests were used. The stabilometric parameters were analyzed also using Kruskal–Wallis test with Dunn’s multiple comparison test, α < 0.05. Data are presented as means ± SD or SE, medians, minimums, maximums, and first and third quartile ranges. The significance has been settled at 0.05.

## Results

### Analysis of General Dynamic Coordination and Postural Parameters with Respect to Motor Quotient Categories

#### Motor Control Skills: The *KTK* Test

In all, 83% of the children regularly practiced sports activities at least twice a week, for a total of 3 h. The *KTK* assessment allowed the children (*n* = 84) to be classified into four different categories based on their MQ scores. The one child who showed a disability (one subject as MQI) was excluded from the subsequent analysis. In summary, the children in the sample (*n* = 83) performed with normal (MQN; *n* = 65; 77% of total) and good (MQG; *n* = 13; 15% of total) motor abilities and mild motor dysfunctions (MQMD; *n* = 5; 6% of total; **Table 1**).

**Table 1:** Motor quotient subcategories and body mass index statistics for the children included in the present study.

MQ sub-category	Children [*n* (%)]	MQ						BMI (kg/m^2^)					


		Mean ± SD	Median	Minimum	Maximum	1 quartile	3 quartile	Mean ± SD	Median	Minimum	Maximum	1 quartile	3 quartile
MQI	1 (1)	–	–	–	–	–	–	–	–	–	–	–	–
MQS	0 (0)	–	–	–	–	–	–	–	–	–	–	–	–
MQMD	5 (6)	81.3 ± 3.2	79	78	85	79	84	22.85 ± 3.79	22.7	18.38	27.54	20	25.6
MQN	65 (77)	101.9 ± 8.1	103	86	115	96	109	18.68 ± 3.26	18.2	14.02	28.12	16.2	19.8
MQG	13 (15)	120.8 ± 4.5	119	116	130	118	123	17.33 ± 2.21	17	15.51	23.07	16	17.6
MQH	–	–	–	–	–	–	–	–	–	–	–	–	–

*MQ, motor quotient; MQI, impossible; MQS, severe motor disorders; MQMD, mild motor dysfunction; MQN, normal; MQG, good; MQH, high; BMI, body mass index.*

We recorded the heights and weights of the 83 children to calculate their BMIs according to the international cut-off of Cole et al. (2000). The BMIs of the male and female children were 19.07 ± 2.44 kg/m^2^ and 18.34 ± 2.25 kg/m^2^, respectively, with no significant differences between them. In agreement with the literature (Lopes et al., 2012), there was significant negative correlation for the MQ of these 83 children with their BMI (*r* = -0.50; *p* < 0.0001). The combined children with normal (MQN) and good (MQG) MQ also showed this significant negative correlation with their BMI (i.e., MQN+MQG vs. BMI: *r* = -0.43; *p* < 0.0001). The BMI of the children with mild dysfunction (MQMD) showed a similar range with respect to those of the MQN and MQG children, although the median BMI and the minimum BMI were >25% higher for the MQMD children vs. the MQN+MQG children, while the maximum BMI of the MQMD children was intermediate between those of the MQN and MQG children (**Table 1**).

#### Stabilometric Test

The correlation analysis revealed that the MQ of all of the children did not correlate with any of the OE and CE stabilometric parameters, even when we distinguished among gender and age (data not shown). However, analysis of the stabilometric parameters showed that the RI and COP–SD in CE condition, were significantly higher for the subcategory of MQMD compared to MQN+MQG children (**Table 2**). The RI parameters for the subcategory of MQMD compared to MQN+MQG children was significantly different also applying the correction for multiple comparison (*p* < 0.05).

**Table 2:** The stabilometric parameters of the children in the MQN+MQG and the MQMD categories.

Test	MQN+MQG						MQMD						*p*-value


	Mean ± SE	Median	Minimum	Maximum	1 quartile	3 quartile	Mean ± SE	Median	Minimum	Maximum	1 quartile	3 quartile	
COP–SD, OE (mm^2^)	71.3 ± 11.4	38.1	3.7	539.5	15.5	74.4	60.4 ± 12	59.1	34.3	102	37.6	68.9	0.18
COP–SD, CE (mm^2^)	70.9 ± 13.9	33.5	3.2	794.9	18.5	82.1	120.4 ± 34.7	112.5	57.7	250.9	65.2	115.6	0.01
RI	119.3 ± 8.2	101.3	13.7	311.9	66.35	156.2	189.2 ± 14.80	173.4	167.9	246.0	168.4	190.2	0.01

*COP, Center of pressure; OE, open eyes; CE, closed eyes; SD, Squared distance; RI, Romberg Index; MQ, motor quotient; MQN, normal; MQG, good; MQMD, mild motor dysfunction; SE, standard error.*

### Assessment of Visual–Motor, Visual–Perceptual, Memory, and Planning Processes

#### Visual–Motor Integration (*VMI*) Test

The MQ did not correlate significantly with the *VMI* index. However, MQMD children performed significantly worse compared to the MQN+MQG children (**Table 3**).

**Table 3:** The *VMI* parameters of the children in the MQN+MQG and the MQMD categories.

Test	MQN+MQG						MQMD						*p*-value


	Mean ± SD	Median	Minimum	Maximum	1 quartile	3 quartile	Mean ± SD	Median	Minimum	Maximum	1 quartile	3 quartile	
VMI standard	104.99 ± 14.1	105	75	134	94	117	92.6 ± 5.32	91	87	100	89	96	0.01

*VMI, visual–motor integration; MQ, motor quotient; MQN, normal; MQG, good; MQMD, mild motor dysfunction.*

#### The Kaufman Assessment Battery for Children for Cognitive Functions (*KABC-II*)

We observed significant positive correlations between MQ and the planning and simultaneous processes subtests (**Table 4**).

**Table 4:** Correlation between motor quotient and *KABC-II* indices.

*KABC-II* parameter	Task/Subtest	*r*	*p*-value
Sequential processes tasks	Number Recall	0.18	0.10
	Word Order	0.16	0.14
Planning processes tasks	Pattern Reasoning	0.3	0.01
	Story Completion	0.12	0.27
Simultaneous processes tasks	Rover	0.15	0.17
	Triangles	0.24	0.02
Subtests	Sequential	0.19	0.08
	Planning	0.24	0.02
	Simultaneous	0.23	0.03

*KABC-II, Kaufman Assessment Battery for Children*.

For the planning processes, the Pattern Reasoning but not the Story Completion task correlated with the MQ. For the simultaneous processes, the Triangles but not the Rover task correlated with the MQ. Moreover, MQMD children performed significantly worse than MQN+MQG in Planning subtest (**Table 5**). This was true for the Pattern Reasoning but not for the Story Completion task.

**Table 5:** The *Kaufman Assessment Battery for Children* in the MQN+MQG and the MQMD categories.

*KABC-II* parameter	Task/subtests	MQN+MQG						MQMD						*p*-value


		Mean ± SD	Median	Minimum	Maximum	1 quartile	3 quartile	Mean ± SD	Median	Minimum	Maximum	1 quartile	3 quartile	
Sequential processes tasks	Number Recall	11.43 ± 3.3	12	2	19	9	14	9.6 ± 3.2	8	7	14	7	12	0.10
	Word Order	11.91 ± 4.1	12	4	19	9	15	9.8 ± 3.3	9	5	13	9	13	0.17
Planning processes tasks	Pattern Reasoning	12.49 ± 4.3	13	3	19	9	16	8.2 ± 3.1	8	4	12	7	10	0.02
	Story Completion	11.5 ± 4.2	11.5	2	19	8.25	15	9.6 ± 4.1	10	6	16	6	10	0.15
Simultaneous processes tasks	Rover	11.0 ± 3.5	10	5	19	8	13.75	9.8 ± 4.9	9	6	18	6	10	0.16
	Triangles	10.9 ± 2.7	11	4	17	9	13	9.4 ± 2.9	8	7	13	7	12	0.12
Subtests	Sequential	110.0 ± 19.8	112	62	152	95.5	122.5	98.4 ± 18.3	88	80	121	88	115	0.12
	Planning	112.5 ± 23.2	112.5	59	157	97	132	93.0 ± 15.1	87	79	112	81	106	0.02
	Simultaneous	105.5 ± 15.2	106	70	143	96.25	115	97.6 ± 20.8	91	82	134	88	93	0.08

*KABC-II, Kaufman Assessment Battery for Children; MQ, motor quotient; MQN, normal; MQG, good; MQMD, mild motor dysfunction*.

### Assessment of Handwriting

#### Praxis of Writing (*BVSCO-2*)

Correlations between the MQ and the praxis of writing were not significant for any of the observed variables. Nor did the MQMD group significantly differed from MQN+MQG.

To study the relation between visual–spatial skill and handwriting, we calculated the correlation between LE praxis and Triangle task (*KABC-II*) *r* = 0.27, *p* = 0.02. We considered the LE praxis task (excluding UNO Praxis and NUMBER Praxis tasks) because it involved only cursive writing.

#### Assessment of Handwriting: Grapho-Motor Skills (*DGM-P*)

The MQ did not correlate significantly with any of the observed variables. Nor did the MQMD group significantly differed from MQN+MQG.

To study the relation between visual–spatial skill and handwriting, we calculated the correlation of Triangle task performance (*KABC-II*) with Speed and Metric Variation (*DGM-P*, best condition). The results showed that Visual–Spatial skill is correlated with Speed, (*r* = 0.32, *p* = 0.005) but not with Metric Variation.

## Discussion

General coordination skills of a group of 83 children were analyzed with the aim to investigate the role of dynamic general coordination in the execution of the graphic gesture patterns. As described in the current literature, about 5–10% of children are affected by disorders of motor coordination (Wilson et al., 2013). Accordingly, the population of children enrolled in the present study showed that 6% of them fell into the MQMD category, including children affected by mild motor dysfunctions, with only one child as MQI (impossible to evaluate), over three-quarters as MQN, and 15% as MQG categories, including children with normal and good motor values, respectively. As a preliminary result, the MQMD children showed similar range of BMI with respect to the MQN and MQG children; thus, even if the increased motor coordination quality (MQ) is associated with decreased BMI (Lopes et al., 2012), our data allow us to exclude that the mild motor coordination dysfunction depends exclusively on BMI.

We analyzed the postural aspects of these children using stabilometric parameters and the link to their motor performance under two different conditions, with OE and CE. Indeed, postural control is a complex process that requires interactions between a number of sensory pathways (e.g., somatosensory, visual, vestibular feedback) and postural responses (Winter, 1995; Westcott et al., 1997; Maurer et al., 2006). These result in the spontaneous sway of the organism (Winter et al., 1998), which is measured as the RI – the percentage ratio of the kinesigram surfaces between these CE and OE conditions – which accounts for the influence of the visual system on the balance control ability. That RI might indicate either an increased spontaneous sway under CE, when the visual feedback is suppressed, or a decreased sway under the OE modality, to compensate for the decrease in other sensory information provided by the visual feedback (Marucchi and Gagey, 1988; Norré, 1990).

Although no correlation was found between MQ and RI, the MQMD children had higher RI, partially confirming our first hypothesis. In our sample, the higher RI depended on the increased values in the parameters belonging to the COP area under CE, which indicated that the visual sensory information in these children has an important compensatory role. Therefore, children with motor problems, as opposed to those with typical development, seem to have a predominance of visual control in the balance. This could compromise the automation of movements that is essential for complex motor tasks, such as handwriting. However, our results showed some weakness as demonstrated by the absence of correlation, probably due to the low number of children.

Even if the *VMI* index and MQ did not correlate, the children with motor difficulties still poorly mastered the task that involved the ability to copy complex geometric forms. Therefore, as we found above, the results partially confirmed our second hypothesis. This result suggests that the visual integration of the graphic gesture patterns is affected by the presence of mild dysfunction of general dynamic coordination.

Our third hypothesis was about the relationships between the MQ and scores in *KABC-II* test, with a number of cognitive functions measured that are supposed to underlie graphical movements. We found positive correlation between MQ and two out of three processes (Planning and Simultaneous) assessed by the *KABC-II* test. Indeed, Sequential process did not correlate with motor coordination. In particular, the Pattern Reasoning task and the Triangles task, which are included in the Planning and Simultaneous processes, respectively, correlated with MQ. Therefore, the reasoning and visuospatial processes appeared to share something with motor performance. The Triangles task involves visual–spatial and specifically visual-constructive abilities; the mental rotation of stimuli is necessary to reconstruct the target figure. The same processes can be partially involved in the motor tasks of *KTK* that require to take into account the spatial relation between the body and the surrounding space while moving.

Moreover, the Pattern reasoning task performance (Planning processes) was lower in children with mild dysfunctions of general dynamic coordination (MQMD) compared to the other combined subgroups. As the Planning subtest requires high level abilities to reason about visual stimuli, we can then suspect that more general cognitive abilities are also involved in MQMD performance.

For the last hypothesis, no correlation was found between MQ and the execution of praxis in handwriting. Also grapho-motor skills did not correlate with MQ.

In addition, unlike all of the above cases, no significant differences were found in the writing indices between children impaired in general dynamic motor coordination (MQMD) and children with normal and good motor performance (MQN and MQG). However, both LE Praxis and speed of handwriting (*DGM-P*) correlated with the Triangles task, which is aimed at assessing visual–spatial skills. Visual–spatial skills therefore were shown to affect both handwriting and motor coordination. Since these two abilities did not correlate, our results suggest that handwriting and motor coordination recruit different aspects of visual–spatial skills. This result has important implications for interventions with MQMD children. Moreover, the observed relationships between impairment in motor coordination and visual–spatial processes makes early screening of motor coordination welcome, to save resources and time, and more importantly, to anticipate and counteract the dysfunction. Altogether, our results, showing a lower performance by MQMD children for the different cognitive tasks, indicate the opportunity to implement the most appropriate strategies for promoting a better prognosis. At the same time, we have to look with caution to the results because the correlations are not very high, and other factors, not investigated in the present study, could play an important role in these processes.

## Conclusion

This study suggests that general motor coordination does not have a direct link with the execution of graphic gesture patterns; indeed, both abilities involve visual–spatial processes.

The children with mild motor dysfunction are lower in visual reasoning abilities with respect to those with normal and good MQ. The measurement of the MQ, which can be performed starting from the age of 5 years, can help to highlight children with mild dysfunction who can then be monitored early in their development, and overall during their school period. Indeed, these results are useful to devise a program for screening and intervention with these children. Another open question is about the relation between general motor coordination and handwriting late during development. To answer this question, longitudinal research is necessary.

For future studies, it will be interesting to investigate more deeply the different aspects of visual–spatial processes involved in general motor coordination and in handwriting.

## Conflict of Interest Statement

The authors declare that the research was conducted in the absence of any commercial or financial relationships that could be construed as a potential conflict of interest.
